# Supplementary material for: Identification of photoperiod-induced specific miRNAs in the adrenal glands of Sunite sheep (Ovis aries)
Source: Front Vet Sci. 2022 Jul 22;9:888207. doi: 10.3389/fvets.2022.888207 (PMC9354845; doi:10.3389/fvets.2022.888207)
Supplement: Supplementary file 1 [file Data_Sheet_1.ZIP › Supplementary file/miRDeep2 file.zip!/miRDeep2 file/Know_Structure/SPLP42A2/oar-mir-411a.pdf]

miRBase precursor : oar-mir-411a
Total read count : 49827
oar-miR-411a-5p read count : 46475
oar-miR-411a-3p read count : 3346
remaining reads : 6

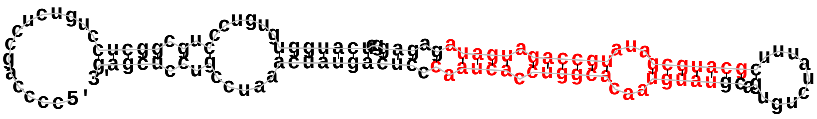

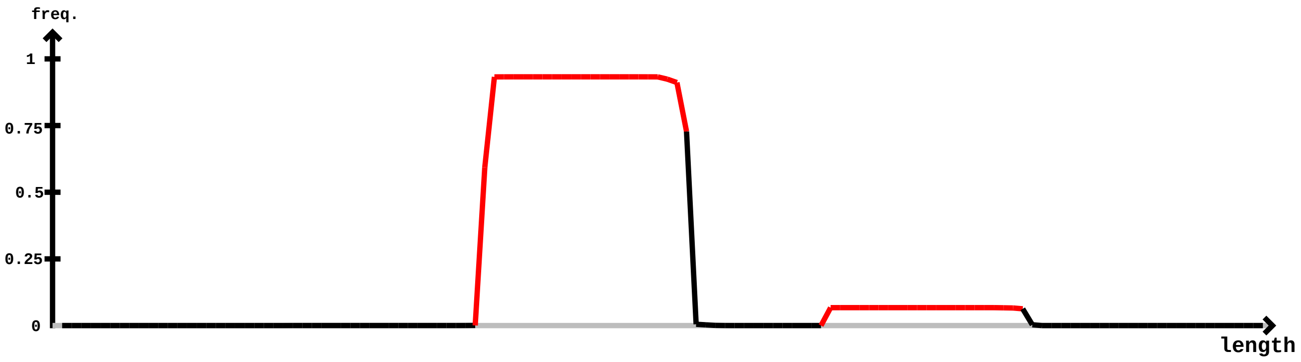


```
                                                                                                   oar-miR-411a-3p
                                          oar-miR-411a-5p
5'- ccccagccucuguccucggcgucccugugugguacucggagagauaguagaccguauagcguacgcuuuaucugugacguauguaacacgguccacuaacccucaguaucaaauccguccucgag  -3'   exp
    ..............(((((.(.(......(((((((..(((.(.((((.((((((...((((((((.........).)))))))...)))))).)))).).))))))))))).....).).)))))   reads     mm
    ......................cccugugugguacucggagag..................................................................................   1         0
    ......................cccugugugguacucggagaga.................................................................................   1         0
    .......................ccugugugguacucggagagau................................................................................   1         0
    ........................cugugugguacucggag....................................................................................   1         0
    ........................cugugugguacucggagag..................................................................................   1         0
    ........................cugugugguacucggagagau................................................................................   1         0
    .........................................agauaguagaccguauagcg................................................................   1         0
    .........................................agauaguagaccguauagcgu...............................................................   2         0
    .........................................agauaguagaccguauagcgua..............................................................   1         0
    .........................................agauaguagaccguauagcguacg............................................................   1         0
    ..........................................gauaguagaccguauagcgu...............................................................   4         0
    ..........................................gauaguagaccguauagcguac.............................................................   3         0
    ..........................................gauaguagaccguauagcguacg............................................................   10        0
    ...........................................auaguagaccguauagc.................................................................   4         0
    ...........................................auaguagaccguauagcg................................................................   7         0
    ...........................................auaguagaccguauagcgu...............................................................   374       0
    ...........................................auaguagaccguauagcgua..............................................................   471       0
    ...........................................auaguagaccguauagcguac.............................................................   5612      0
    ...........................................auaguagaccguauagcguacg............................................................   23019     0
    ...........................................auaguagaccguauagcguacgc...........................................................   48        0
    ...........................................auaguagaccguauagcguacgcu..........................................................   24        0
    ............................................uaguagaccguauagcgu...............................................................   41        0
    ............................................uaguagaccguauagcgua..............................................................   165       0
    ............................................uaguagaccguauagcguac.............................................................   3537      0
    ............................................uaguagaccguauagcguacg............................................................   12967     0
    ............................................uaguagaccguauagcguacgc...........................................................   63        0
    ............................................uaguagaccguauagcguacgcu..........................................................   75        0
    ............................................uaguagaccguauagcguacgcuu.........................................................   26        0
    ............................................uaguagaccguauagcguacgcuuu........................................................   3         0
    .............................................aguagaccguauagcguacg............................................................   6         0
    .............................................aguagaccguauagcguacgcu..........................................................   6         0
    .............................................aguagaccguauagcguacgcuuu........................................................   1         0
    ..............................................guagaccguauagcguac.............................................................   1         0
    ..............................................guagaccguauagcguacg............................................................   2         0
```

```
                                                                               oar-miR-411a-3p
                                           oar-miR-411a-5p
ccccagccucuguccucggcgucccugugugguacucggagagauaguagaccguauagcguacgcuuuaucugugacguauguaacacgguccacuaacccucaguaucaaauccguccucgag

...............................................uagaccguauagcguacg............................................................   1      0
..............................................................................guauguaacacgguccacuaac.........................   1      0
...............................................................................uauguaacacgguccacu............................   26     0
...............................................................................uauguaacacgguccacua...........................   38     0
...............................................................................uauguaacacgguccacuaa..........................   151    0
...............................................................................uauguaacacgguccacuaac.........................   2980   0
...............................................................................uauguaacacgguccacuaacc........................   148    0
...............................................................................uauguaacacgguccacuaaccc.......................   1      0
................................................................................auguaacacgguccacuaac.........................   1      0
```
